# Indocyanine-Green-Guided Partial Nephrectomy: A Narrative Review Addressing Protocol Heterogeneity, Perioperative Functional and Oncological Outcomes

**DOI:** 10.3390/medicina62020347

**Published:** 2026-02-09

**Authors:** Vlad Cristian Munteanu, Raluca Munteanu, Răzvan Crețeanu, Alexandru-Florin Badea, Carmen Bianca Crivii

**Affiliations:** 1 Anatomy and Embryology Discipline, Morphological Sciences Department, Iuliu Hațieganu University of Medicine and Pharmacy, 400000 Cluj-Napoca, Romania; vladcristian.munteanu@gmail.com (V.C.M.); creteanurazvandrc@yahoo.com (R.C.); afbadea84@gmail.com (A.-F.B.); 2 Department of Urology, “Prof. Dr. Ion Chiricuta” Oncology Institute, 400015 Cluj-Napoca, Romania; 3 Department of Personalized Medicine and Rare Diseases, Medfuture Institute for Biomedical Research, Iuliu Hațieganu University of Medicine and Pharmacy, 400337 Cluj-Napoca, Romania; muresan.raluca.andrada@gmail.com

**Keywords:** partial nephrectomy, NIRF imaging, ICG, nephron-sparing surgery, selective clamping, fluorescent-guided tracer

## Abstract

*Background and Objectives:* Partial nephrectomy is the preferred intervention for many localized renal tumors; but intraoperative tumor localization, real-time perfusion evaluation, and vascular control optimization can be technically demanding, especially in endophytic or complex lesions. Near-infrared fluorescence (NIRF) imaging with indocyanine green (ICG) has been adopted as an intraoperative adjunct to improve visualization, support selective or super-selective clamping strategies and assist tumor to parenchyma contrast and selective vascular control. However, current evidence regarding the benefit of ICG-NIRF is often inconsistent, and a significant gap exists due to the lack of standardized intraoperative protocols, which limits the reproducibility of clinical results. This review aims to synthesize existing comparative evidence, identify the sources of methodological heterogeneity, and propose minimum criteria for the standardization of ICG use in renal surgery. *Materials and Methods:* A narrative review was conducted using PubMed with the terms near-infrared fluorescence, indocyanine green, and partial nephrectomy, focusing on comparative clinical studies published since 2012. Key endpoints included warm ischemia time (WIT), positive surgical margins (PSMs), perioperative outcomes, short-term renal functional measures (eGFR and or split renal function), and available oncologic follow-up. *Results:* ICG-NIRF enables real-time visualization of renal perfusion and vascular anatomy and may improve tumor parenchyma contrast in superficial or partially exophytic tumors, facilitating selective clamping in selected cases. Comparative cohorts and meta-analyses report small reductions in WIT (approximately 1 to 3 min) in some series, modest short-term superiority in eGFR (e.g., 4.62 mL/min at discharge or 9.26 mL/min at 1 to 3 months), no consistent differences in PSM rates (reported ranges of 0 to 11 percent across studies), major complications, or recurrence outcomes. Durable improvements in long-term renal function and consistent benefits in split renal function have not been demonstrated. Interpretation is limited by heterogeneity in ICG dosing, timing, imaging platforms, and acquisition. *Conclusions:* ICG-NIRF is a useful adjunct for intraoperative perfusion assessment and selective vascular control during partial nephrectomy, but current evidence does not demonstrate long-term functional or oncologic benefit over standard approaches. Further progress requires protocol standardization, quantitative fluorescence metrics, and adequately powered trials with long-term functional and oncologic endpoints, together with the development of deeper-penetrating and more tumor-specific fluorophores.

## 1. Introduction

Partial nephrectomy is generally considered the optimal approach for managing most clinically localized renal tumors, as it allows for the preservation of kidney tissue while providing cancer control equivalent to that achieved by radical nephrectomy in carefully selected patients. The primary objectives include achieving negative resection margins, reducing the duration of warm ischemia, and maintaining postoperative renal function. These outcomes are often evaluated using composite measures such as the “trifecta” (negative margins, acceptable warm ischemia time, and absence of major complications) and the “pentafecta” (which adds preservation of more than 90% of the estimated glomerular filtration rate and no progression to higher stages of chronic kidney disease at follow-up) [1].

Although minimally invasive and robotic surgical techniques have made substantial progress, particular intraoperative challenges still exist. Accurate localization of tumors, especially those that are endophytic or centrally situated as well as preserving precise vascular control and reliable real-time assessment of parenchymal perfusion, continue to present significant difficulties [2].

The introduction of near-infrared fluorescence (NIRF) imaging using indocyanine green (ICG) has provided an intraoperative adjunct for addressing several technical challenges in partial nephrectomy. When administered intravenously, ICG binds quickly to plasma proteins and remains primarily within the vasculature, emitting fluorescence in the near-infrared spectrum. This property allows for direct, real-time visualization of renal perfusion and vascular anatomy without exposing the patient to ionizing radiation [3]. In clinical practice, ICG can improve the distinction between tumor and normal kidney tissue, facilitate selective or highly targeted vascular clamping, and offer immediate information regarding tissue perfusion throughout and following tumor excision. These advantages have led to the adoption in some cases of NIRF imaging in renal surgery. Many centers have incorporated this technique into their routine practice, but the reported impact on perioperative variables such as warm ischemia time and blood loss, as well as postoperative renal function, shows considerable variability in the literature. Moreover, there does not appear to be a significant difference in oncologic outcomes when compared to traditional techniques [4].

A primary impediment to reaching final conclusions is the substantial methodological variability observed across studies. There are notable differences in dosing strategies (such as fixed versus weight-based dosing), the timing of ICG administration in relation to vascular clamping, protocols for repeat dosing, the type of imaging platforms used, camera distance from the surgical field, exposure settings, and the influence of ambient lighting. This heterogeneity makes the comparison between studies difficult and limits the reliability of meta-analyses [5,6,7].

This review synthesizes the chemistry and pharmacology of ICG relevant to renal surgery, summarizes optical and acquisition parameters that influence image quality, and provides current comparative evidence for ICG-guided partial nephrectomy.

We performed comprehensive literature research through the PubMed/MEDLINE and Google Scholar databases for articles published from January 2012 until December 2025. The PubMed research combined MeSH terms and phrases, specifically: “near-infrared fluorescence”, “indocyanine green”, “ICG”, and “partial nephrectomy”. AND, OR were utilized to enhance the results (e.g., “indocyanine green” AND “partial nephrectomy”). We incorporated comparative papers, including retrospective, prospective, and randomized trials. We excluded studies that indicate the feasibility of ICG or are non-comparative. The preliminary evaluation was conducted based on the relevance of titles and abstracts. Subsequently, full-text papers were obtained and assessed for eligibility. In Sectoin 4, we focus on comparing clinical/functional outcomes and oncological safety.

## 2. ICG Chemistry and Pharmacology

Indocyanine green (ICG) is a water-soluble tricarbocyanine dye with a molecular weight of 775 Da that exhibits peak absorption at 780–805 nm and fluorescence emission at 820–840 nm, making it suitable for near-infrared tissue imaging. Over 95% of intravenously administered ICG binds rapidly and reversibly to plasma proteins, predominantly albumin. This aspect restricts its distribution to the intravascular compartment and supports its effectiveness in angiographic and lymphangiographic procedures [7].

The dye’s high affinity for plasma proteins contributes to its hepatic clearance, since ICG is actively absorbed by hepatocytes (mainly via OATP1B1/1B3 and NTCP transporters) and excreted unchanged into the bile within 10 to 15 min after injection, with minimal renal elimination, even in cases of severe renal impairment. The plasma half-life is typically 3 to 5 min in healthy individuals [8].

According to the perioperative imaging literature, indocyanine green (ICG) is supplied as a lyophilized powder, often stabilized with sodium iodide, and is typically reconstituted to a concentration of 2.5 mg/mL (for instance, dissolving 25 mg in 10 mL of sterile water). Prepared solutions are protected from light and, in most institutional protocols, are used up to a few hours after preparation [9].

Intraoperative series typically describe intravenous bolus administration, which is frequently followed by a small saline flush to achieve rapid intravascular delivery. Dosing in these reports clusters around 0.1–0.5 mg/kg, with occasional use up to 1 mg/kg, and cumulative perioperative amounts generally remaining ≤ 2 mg/kg. The fluorescence response is concentration-dependent; protein binding leads to increased signal, while elevated local dye concentrations may induce aggregation and self-quenching effects [10]. Although earlier guidance advised caution in patients with so-called “iodine hypersensitivity”, current evidence indicates no cross-reactivity between iodine, shellfish, or iodinated contrast allergies and indocyanine green [11,12].

The key contraindication is a known hypersensitivity to indocyanine green [13]. Intravenous ICG is generally well tolerated up to a total dose of 2 mg/kg, and most perioperative protocols use approximately 0.1 to 0.5 mg/kg (occasionally up to 1 mg/kg) for optimal imaging. Allergic reactions are rare, with an incidence of about 0.05% for serious events, but anaphylaxis has been reported, so resuscitation capability should be immediately available during administration [14,15].

In summary, the utility of ICG is a direct result of its biochemical properties. It almost exclusively binds to plasma proteins and remains trapped inside the blood vessels, which transforms the vascular system into a fluorescent map, and helps surgeons distinguish between perfused and devascularised areas in real time. The hepatic clearance is especially benefic for partial nephrectomies because it is not urinarily excreted, and it remains safe even for patients with renal insufficiency. ICG acts as a light emitting biomarker that provides high-contrast tissue perfusion without systemic retention.

## 3. Optical Properties Relevant to Partial Nephrectomy

### 3.1. Spectral Characteristics

ICG exhibits a principal NIR absorption ~780–805 nm and emission ~820–840 nm [16,17]. Both the absorption and emission maxima of ICG are sensitive to environmental conditions, such as solvent composition, temperature, and ionic strength [16]. Among the most influential factors is the interaction with serum proteins or lipids, which not only stabilizes the dye but also generally boosts fluorescence intensity and induces a slight red shift in the emission peak [16].

At higher concentrations, ICG forms H-aggregates (blue-shifted) and J-aggregates (red-shifted), promoting self-quenching and reducing effective signal; in proteinaceous media, intensity often peaks at ~10–30 µg/mL and declines beyond this range. As a result, careful perioperative handling that prevents excessively high local concentrations is important for maintaining optimal imaging sensitivity [18,19].

### 3.2. Intraoperative Applications of ICG-NIRF in Partial Nephrectomy

For intraoperative use in partial nephrectomy, ICG provides real-time, radiation-free visualization of tumor visualization, tissue perfusion and vascular anatomy during both open and minimally invasive procedures, including robotic interventions [20,21].

- *Tumor visualization and tumor parenchyma contrast*

ICG-NIRF provides tissue differentiation, normal renal tissue accumulates dye compared with renal tumors which lack bilitranslocase (plasma membrane protein) and therefore lack dye accumulation and tumor visualization [22].

B. *Perfusion assessment including ischemia and reperfusion mapping*

Following intravenous bolus administration, ICG binds rapidly to plasma proteins and remains largely intravascular, producing a detectable near-infrared (NIR) signal within minutes on clinical systems. Clinically, ICG perfusion assessment is widely used in colorectal, vascular, plastic, and transplant surgeries to confirm adequate blood supply to anastomoses, flaps, or grafts and to guide intraoperative decision making [23]. ICG-guided perfusion assessment has demonstrated utility in optimizing surgical strategy, improving identification of renal vessels, and facilitating selective arterial clamping, which can reduce blood loss and preserve renal function. Tumors typically appear hypofluorescent compared to surrounding normal parenchyma, aiding in precise tumor excision while sparing healthy tissue [24]. It can help tailor intraoperative strategy, confirm adequate reperfusion, and potentially reduce the risk of postoperative renal dysfunction by identifying areas of hypoperfusion that may require further intervention [25].

C. *Selective and super-selective vascular clamping with targeted ischemia*

Another application of ICG in renal tumors is aiding in supraselective clamping of renal vessels. Intraoperatively, after renal hilum dissection, the surgeon continues along the segmental arteries and clamps the one that provides blood to the tumor. After clamping, when ICG is injected, the clamped area remains without dye. This technique in theory should maximize renal preservation [26].

Quantitative fluorescence measurements are most reproducible when imaging geometry is carefully controlled. Many surgical NIR imaging platforms are optimized for working distances of approximately 30 cm, balancing field of view with ergonomic considerations [27] As the distance between the camera and the target increases, observed fluorescence intensity typically decreases. In some laparoscopic systems, this decline approximates an inverse-square relationship, although the specific pattern depends on device- and setting-related factors such as illumination geometry, gain, and auto-exposure settings [21,28]. The imaging protocol is simple and allows immediate intraoperative assessment of perfusion, tumor margins, and anatomical reference points, without ionizing radiation [29].

### 3.3. Penetration Depth and NIR-II

The effective tissue penetration depth of ICG NIR-I fluorescence is limited to a few millimeters (often ~2–5 mm and at most ~1 cm, depending on tissue optics and the imaging system), which represents a considerable technical limitation to the identification of deep or endophytic lesions [30,31].

NIR-II fluorescence imaging (1000–1700 nm) reduces scattering and autofluorescence, resulting in increased penetration and superior contrast in comparison to traditional NIR-I [32]. However, clinical applications are still developing, and there are few dedicated, widely approved NIR-II probes [33].

### 3.4. Imaging Geometry, Working Distance, and Ambient Light

Fluorescence intensity decreases as camera-to-target distance increases, and in some laparoscopic systems this approximates an inverse-square relation, although the exact falloff is device- and setting-dependent (illumination geometry, gain, auto-exposure) [5,34].

In practice, many surgical NIR systems are commonly operated at a ~30 cm working distance to balance field-of-view and ergonomics, whereas quantitative perfusion work sometimes may favor closer positioning (~4–5 cm) to maximize signal to noise. So, when comparing fluorescence intensities during surgery, teams should use the same distance and exposure for the cameras [35,36].

Also, ambient surgical lighting can interfere with fluorescence detection, requiring lowered light or specialized filtering systems to optimize signal visualization [37].

### 3.5. Intrarenal Fluorescence Distribution

In renal tissue, ICG being intravascular reflects perfusion. Because cortical blood flow significantly exceeds medullary flow, the cortex typically appears more fluorescent than the medulla. This physiology helps interpret normal renal fluorescence patterns during partial nephrectomy, although formal intraoperative quantification of cortex-versus-medulla intensity is limited [38]. In partial nephrectomy, this physiological distribution is important for analyzing global reperfusion postunclamping and for evaluating the degree of segmental ischemia during selective or super-selective clamping [3,39]. It is also significant for analyzing fluorescence within the resection bed. Decreased fluorescence in the deeper areas of a resection bed should be evaluated with caution, since signal attenuation may indicate restricted NIR-I penetration rather than actual hypoperfusion [40]. Accordingly, fluorescence should be considered supplementary information in conjunction with surgical context and controlled acquisition settings, rather than as an independent indicator of deep tissue perfusion.

## 4. Partial Nephrectomy

In partial nephrectomy, the primary objectives are to achieve negative surgical margins and minimize warm ischemia time while preserving maximal renal function. These goals are frequently summarized by combined results such as the trifecta (negative margins, WIT within a predefined threshold commonly ≤20–25 min and absence of major complications) and the pentafecta (trifecta plus >90% eGFR preservation and no CKD stage upstaging at 6–12 months) [41,42].

Reported achievement rates vary widely with tumor complexity, definitions, and surgical approach. Prolonged warm ischemia (>25–30 min) is associated with worse short-term renal function, although the level of reduction depends on baseline renal function, the amount of preserved parenchyma, and whether cold ischemia is used [43]. ICG fluorescence may support these goals by improving lesion parenchyma contrast, improving selective vascular control, and facilitating efficient tumor identification, which can help shorten ischemia time [44].

Trifecta and pentafecta outcomes integrate oncologic adequacy, ischemia quality, complications, and functional preservation and therefore provide a clinically useful framework to interpret the ICG literature [45]. Based on Table 1 and pooled analyses, ICG guidance appears most likely to influence the ischemia component of the trifecta, primarily by supporting selective clamping and achieving modest reductions in warm ischemia time in some series [6,7,40,41,44]. In contrast, positive surgical margin rates and major complication rates are generally similar between ICG-guided and standard partial nephrectomy, suggesting limited additional impact on the oncologic and safety components of the trifecta [6,7,40,41]. Regarding the pentafecta, several comparative cohorts report small early differences in postoperative eGFR, but durable long-term functional benefit and consistent improvement in split renal function have not been demonstrated, which limits the evidence that ICG increases pentafecta achievement.

### 4.1. Diagnostic Discrimination

ICG-NIRF is used for intraoperative tumor visualization (tumor–parenchyma contrast), but fluorescence patterns do not reliably characterize histology. In the most extensive validation to date, Manny et al. [46] prospectively assessed 100 robot-assisted partial nephrectomy cases with Firefly fluorescence and reported a sensitivity of 84%, specificity of 57%, positive predictive value of 87%, and negative predictive value of 52% for malignancy prediction performance comparable to, but not superior to, existing imaging techniques. All cystic lesions and angiomyolipomas were consistently afluorescent, while 96% of clear-cell renal cell carcinomas appeared hypofluorescent; however, 18 benign solid tumors, including oncocytomas, exhibited hypofluorescent patterns identical to malignancies, underscoring that ICG fluorescence reflects vascular perfusion rather than histological features [46]. This diagnostic overlap is particularly concerning given that contemporary surgical series demonstrate benign pathology in ~20–30% of resected small renal masses [47,48]. Moreover, ICG’s diagnostic performance does not exceed that of CT or MRI, which achieve sensitivities of ~76–92% and specificities of ~63–88% for T1 renal mass malignancy detection without the technical constraints of fluorescence imaging [47]. Therefore, ICG is a valuable intraoperative adjunct for visualization and vascular control, but it cannot replace preoperative cross-sectional imaging for surgical planning; fluorescence phenotype alone should not guide tumor characterization or treatment decisions without correlation to preoperative imaging and pathology.

### 4.2. Clinical Outcomes (Comparative Evidence)

Multiple comparative studies have evaluated the impact of ICG fluorescence guidance on key surgical and functional outcomes in partial nephrectomy.

A prospective comparative study which evaluated ICG in robotic partial nephrectomy analyzed 94 patients (47 with ICG guidance and 47 standard), found no significant difference in the rate of positive surgical margins (three vs. four cases). However, the ICG group had a slightly shorter warm ischemia time (15 vs. 17 min), a difference of 2 min whose clinical relevance is uncertain when ischemia times are within accepted thresholds. Estimated blood loss was slightly higher in the ICG group (75 vs. 50 mL), however, perioperative hemoglobin change was similar, and transfusion was uncommon (zero in the ICG group vs. one in controls). Additionally, the presence of green fluorescence at the tumor margin may indicate residual normal renal tissue within the excised specimen [4].

A systematic review including six studies with a total of 369 patients evaluated operative times, estimated blood loss, warm ischemia time, postoperative complications, positive surgical margins, eGFR decline at discharge, eGFR value at 1–3 months and split kidney function. Shorter ischemia time with NIRF was identified only in one study (difference of 1.4 min); no significant differences were found in postoperative complications, positive margins, or eGFR at discharge (4.62 mL/min, *p* = 0.26), but at 1–3 months eGFR was slightly better in the NIRF group (9.26 mL/min, *p* < 0.001) [49].

A subsequent systematic review and meta-analysis reported similar pooled results showing no difference (operating time, estimated blood loss, complications, transfusions, positive margins) except the eGFR at discharge (standardized mean difference 0.44) and shorter ischemia time for the NIRF group (mean difference—1.4, *p* = 0.001) [6].

Consistent findings were reported again, with no significant differences between groups in terms of operating time, estimated blood loss, length of stay, major or minor complications, urinary fistulas, blood transfusion rate, positive margins or tumor recurrence, but postoperative eGFR (weighted mean difference 7.67) is in favor of the ICG group [7].

Despite these perceived benefits, the utility of ICG fluorescence in partial nephrectomy is limited by several technical and biological factors. For example, the NIR-I penetration of ICG is limited to only a few millimeters beneath the renal capsule. As a result, completely endophytic tumors, intrarenal bell-shaped extensions, or small venous thrombi cannot be adequately assessed intraoperatively using ICG fluorescence [50].

Dosing is another critical determinant of imaging reliability. Underdosing of ICG may produce weak fluorescence of normal renal parenchyma, creating ambiguity in differentiating tumor margins. On the other hand, overdosing can cause uniform hyperfluorescence of the entire renal parenchyma, which tends to obscure the contrast between the tumor and surrounding tissue [30,44]. To address this, some authors have proposed a two-step approach by administering a small test dose followed by titration in order to optimize fluorescence intensity and avoid both under- and oversaturation [30].

This method was tested on a prospective study on 79 patients, for which the ICG test dose was a median of 1.25 mg (ranging from 0.625 to 2.5) and the calibration dose was 1.875b (0.625 m to 5 mg). The test dose was administered; the kidney and the tumor aspect were inspected through NIRF. As the fluorescent dye washed, the surgeon prepared for the next steps and, immediately before clamping, administered the calibrated dose to block the dye in the kidney and not have it washed away. This helped with tumor identification during the excision and also provided a green tumor bed for negative margins (in case of differential coloring of the tumor) As the fluorescent dye washed out, the surgeon prepared for the next steps and, immediately before clamping, administered the calibrated dose to maintain renal fluorescence during excision. This helped with tumor identification during the excision and allowed NIRF assessment of the resection bed. In cases where the tumor appeared hypofluorescent relative to parenchyma, a uniformly green resection bed was consistent with preserved, well-perfused renal tissue, whereas a focal hypofluorescent area in the bed raised concern for possible residual tumor at the margin [51].

An alternative method is to identify and secure the renal artery, clamp it and inject 3–5 mL of ICG dye (25 mL ICG in 10 mL distilled water) intra-arterially, and observe arterial illumination at about 75 s; then the clamp is briefly released to permit parenchymal filling and then reapplied to limit washout [52].

Multiple studies have explored the impact of ICG guidance on outcomes in partial nephrectomy; results are synthesized in Table 1.

**Table 1:** Comparative Outcomes of ICG-Guided Versus Standard Partial Nephrectomy.

Study (Year)	Design	Margins (PSM)	WIT (min)	eGFR Change/Follow-Up (mL/min/1.73 m^2^)			
				Discharge	1 Month	3 Months	6 Months
Krane et al. (2012) [4]	Pr	NS (6% vs. 8.5%)	15 vs. 17 (*p* = 0.03)	NS (−4.6% vs. −7.4%)	NE	NE	NE
Katsimperis et al. (2024) [40]	SR	NS (0–11%)	Shorter (3–4)	Small loss (NS)	NE	NE	NE
Yang et al. (2022) [52]	R	NS (11% vs. 8%)	NS (21 vs. 24)	NS (81 vs. 64)	NE	90% vs. 85% (*p* = 0.03)	NS
Veccia et al. (2020) [49]	SR/MA	NS	Shorter with ICG	NS (MD 4.62)	Favors ICG (MD 9.26)	Favors ICG (MD 9.26)	NE
Zhou et al. (2023) [6]	SR/MA	NS	Shorter (MD −1.4)	Favors ICG (SMD 0.44)	NS	NS	NE
Giulioni et al. (2023) [7]	SR/MA	NS	Shorter with ICG	Favors ICG (WMD 7.67)	NE	NE	NE
Panunzio et al. (2025) [50]	SR/MA	NS	Shorter with ICG	NS	NS	NS	NS
Joffe et al. (2025) [53]	R	NS	NS	NS	NS	NS	NS

Abbreviations: SR/MA: Systematic Review/Meta-analysis; R: Retrospective; Pr: Prospective; NE: Not Evaluated; NS: Not Statistically Significant (*p* > 0.05); MD: Mean Difference; SMD: Standardized Mean Difference; PSM: Positive Surgical Margin; WIT: Warm Ischemia Time.

A major area of ongoing debate involves the clinical impact of ICG-guided selective or super-selective clamping for nephron preservation during PN. The EMERALD randomized single blind trial is currently the only randomized controlled trial evaluating super-selective ischemia with ICG guidance and therefore represents the highest-level evidence for functional preservation claims in this setting. The study was terminated early after enrolment of 30 patients following interim analysis at 6-month follow-up. The prespecified functional endpoints, including estimated glomerular filtration rate and relative renal function assessed by 99mTc-DMSA scintigraphy, showed no significant improvement compared with standard renal artery clamping techniques [26]. As a randomized trial, EMERALD provides the most reliable estimate of effect and should be weighed more heavily than favorable signals from retrospective matched or cohort studies, which are more susceptible to selection bias and residual confounding.

These findings raise questions about the clinical utility of ICG-guided super-selective clamping, particularly given the added technical complexity and potential vascular risks associated with this method. Possible benefits may exist in anatomically challenging cases, such as a large tumor with more complex and accessory vascularization, or in patients with solitary or pelvic kidneys, as well as in those with impaired renal function. However, despite reported correlations between ICG and trifecta achievement, no consistent functional advantage has been described by most surgeons [3,6,7].

Beyond EMERALD, the ICG-specific comparative evidence remains heterogeneous. Observational matched and cohort studies of ICG-guided selective or super-selective clamping have reported short-term functional gains, including higher discharge eGFR and smaller six-month eGFR declines in some series [52].

Postoperative renal function after partial nephrectomy is driven primarily by the amount and quality of preserved renal parenchyma, with warm ischemia time showing a smaller additional effect when kept within commonly accepted thresholds. Multicenter analyses have shown that preserved parenchymal volume and preoperative renal function are the dominant predictors of long-term renal outcomes, whereas the contribution of ischemia duration becomes most apparent when warm ischemia is prolonged beyond approximately 25 min [54]. Moreover, in contemporary partial nephrectomy series, positive surgical margin rates are generally low. In a systematic review and meta-analysis of comparative studies, positive surgical margins occurred in 6.7% of cases overall, with a mean estimated rate of approximately 7%, including about 7% in robot-assisted series. At these baseline rates, any additional absolute reduction attributable to ICG guidance is likely to be small and may be difficult to detect in heterogeneous and often underpowered comparative cohorts [55].

## 5. Discussion, Limitations and Standardization

The current evidence bases for ICG use in partial nephrectomy is promising but methodologically limited and heterogenous. The interpretation of current data is subject to several limitations. First, the high heterogeneity of included studies, non-randomization, ranging from small retrospective cohorts and case series to systematic reviews, lacking standardized controls, and introducing selection and confounding biases. The benefits seem to be short-term and heterogenous, and for long-term and consistent results, split renal function should be performed. Second, the lack of standardized ICG protocols makes it difficult to determine if a lack of benefit is due to the technology itself or to sub-optimal dye administration. Third, NIR-I technology is limited by tissue penetration depth (2–5 mm), which restricts its generalizability to deeply endophytic tumors. Furthermore, most comparative results stem from high-volume academic centers, which may not reflect the outcomes achievable during the initial learning curve in lower-volume settings. Table 2 summarizes the principal protocol parameters, typical ranges, and key observations reported across existing studies.

When interpreting the results, the superiority of WIT (1–3 min) and eGFR in the NIRF-ICG group should be cautiously interpreted because most of the studies did not reach statistical significance and the translation into day-to-day workflow may not reach a clinical impact, as long as the WIT is in acceptable parameters (20–25 min) [1]. Also, superior eGFR at discharge represents only a temporary benefit which should be interpreted with caution.

Giulioni et al. state that, even though ICG is valuable in tumor identification and vascularization, their data indicates that a successful RAPN with maximal functional outcomes depends on the 3D image, robotic system, and precise dissection [7].

Partial nephrectomies represent one of the most complex surgeries in urology, with tumors that can vary in complexity, for which different scores have been created (PADUA, RENAL, etc.). They can raise the risk of bleeding, urinary fistula, or other intraoperative complications including conversion to open surgery or radical nephrectomy [56]. Only experienced surgeons perform nephron-sparing surgeries, and they should begin with tumors with a PADUA (RENAL) score < 7. Experienced surgeons can achieve the trifecta in complex tumors, with low complications. This type of operation should only be performed in high-volume centers [57]. Selective clamping is another variable, usually chosen for less complex tumors [58] (but the lack of standardization, often converging to global clamping, interferes with the true results of this technique [59]). All of the above represent a risk of bias which should be accounted for when choosing the operating plan for a renal tumor.

In regard to oncological safety, there is no long-term follow-up specific for NIRF-ICG-operated patients since the rates of positive and negative margins are the same in both groups, so a significant difference in oncological survival is not expected.

ICG protocols vary substantially in dose (e.g., 0.25 to 0.5 mg/kg bolus vs. fixed 0.625 to 7.5 mg), timing (immediately before clamping to several minutes prior), and redosing (single vs. multiple boluses) [60].

Imaging parameters such as camera working distance, illumination, filter configuration, and NIR-I vs. NIR-II platform are inconsistently specified, as are outcome definitions and assessment intervals for warm ischemia time, estimated blood loss, and postoperative eGFR change [7].

Consistent with these concerns, Katsimperis et al. screened 522 records and included 14 studies for qualitative synthesis only. Most series used a 5 mg dose of indocyanine green; reported outcomes were highly variable: warm ischemia time ranged from 11.6 to 27.2 min, the decrease in estimated glomerular filtration rate ranged from 0 to 15.47 percent, estimated blood loss ranged from 48 to 347 milliliters, and positive surgical margin rates ranged from 0 to 11 percent. In the few comparative cohorts, warm ischemia time was shorter with indocyanine green in some groups (for example, 21.33 versus 25.33 min and 16.3 versus 19.66 min, with a *p* value less than 0.001), and the rest of the outcomes matched earlier studies. Using a structured risk of bias assessment, the study concluded there was moderate to serious bias due to selection, protocol variability, small sample sizes, and incomplete follow-up and therefore deliberately avoided meta-analysis, showing the need for standardized protocols and higher-quality comparative trials. They also observed that selective clamping presented (for which ICG was used) lower eGFR loss compared with global ischemia (6.2% vs. 15.4%) [40].

**Table 2:** Heterogeneity in Evidence (comparative partial nephrectomy studies).

Parameters	Typical Ranges/Variability	Observations	References
Dose (per bolus)	0.1–0.5 mg/kg (occasionally to 1 mg/kg); fixed 2.5–7.5 mg when using 2.5 mg/mL	Report concentration (mg/mL) and total mg; many studies keep cumulative dose ≤ 2 mg/kg	[30,60]
Timing	Immediately preclamping, during arterial mapping, or postresection	Study- and strategy-dependent	[4]
Redosing	Single vs. multiple boluses	Often ≤ 3, but varies by signal need and protocol	[61]
Imaging platform	NIR-I standard; NIR-II in select reports	Filter/camera variance common; platforms rarely standardized across studies	[33]
Acquisition conditions	Working distance ~30 cm (clinical), closer for quantitative work; ambient OR light varies	Distance, exposure/gain, and ambient light are often unreported but materially affect signal	[5,62]
WIT	11.6–27.2 min	Broad range; small, pooled reductions with ICG in meta-analyses	[4,40]
EBL	~48–347 mL	Highly variable, no consistent pooled difference	[40]
eGFR change	0–15.47% decrease (early timepoints)	Signals of short-term benefit with ICG; longer-term differences inconsistent	[7,40]
PSM rate	0–11%	Low overall; definitions and follow-up vary	[40]

In addition to technical and reporting heterogeneity, patient-related factors also limit generalizability of ICG-guided PN. For example, in pregnant patients, human data in pregnancy are limited, and older maternal–fetal sampling studies did not detect placental transfer, while preclinical mouse imaging demonstrates fetal and placental exposure that can increase with concomitant transporter inhibitors. In these cases, ICG should be used in pregnancy only if clearly indicated, after individualized risk–benefit assessment and consideration of potential drug–drug interactions [63,64].

Pediatric applications require careful consideration of weight-based dosing (typically ~0.25–0.5 mg/kg IV, with ~0.3 mg/kg commonly used for renal procedures), with developing evidence from small series suggesting feasibility in children but limited long-term safety data [65,66]. Also, elderly patients may present altered ICG pharmacokinetics due to decreased hepatic function, potentially prolonging fluorescence duration and affecting imaging interpretation. Most critically, patients with significant hepatic impairment experience delayed ICG clearance, leading to prolonged systemic retention, altered fluorescence patterns, and potential accumulation with repeated dosing factors that may compromise both safety and imaging reliability [67].

## 6. Future Perspectives

Although ICG fluorescence has improved intraoperative visualization and perfusion assessment during partial nephrectomy, several important challenges remain before it can achieve its full clinical potential. Future advances can be organized into four priority areas: protocol standardization, quantitative imaging with computational support, development of next-generation fluorophores, and context-specific application in special populations.

First, it is important to establish and adopt evidence-based protocols that address dosing, timing, redosing, and imaging parameters. Consensus-based reporting standards are essential to reduce methodological variability, support reliable meta-analyses, and facilitate regulatory approval and cost coverage across diverse healthcare systems [7].

Second, the transition from primarily qualitative assessment to quantitative fluorescence analysis may improve reproducibility and clinical decision making. Quantitative approaches such as signal-to-background ratios and predefined cut-offs, ideally supported by algorithmic or artificial-intelligence-based tools for objective measurement, could standardize interpretation across centers and platforms. Coupled with improved device calibration, cross-platform harmonization, and real-time quantitative feedback, these methods could transform ICG from a subjective visual aid into a dependable intraoperative metric [68,69]. To strengthen the prospective value of NIRF-ICG imaging and improve comparability across studies, we propose a minimum harmonization framework focused on protocol reporting and acquisition control. At minimum, studies should standardize (or explicitly report) ICG dose strategy (mg/kg vs. fixed dose), concentration and total administered dose, and the exact timing schema (bolus-to-imaging interval, bolus-to-clamping interval, and any repeat dosing), because fluorescence signal is highly dependent on these parameters. Imaging acquisition should be performed under controlled and reproducible conditions, including the specified NIR platform, camera-to-target working distance, illumination conditions/ambient light management, and fixed exposure/gain settings where possible, as these variables materially influence measured fluorescence intensity and interpretation. Also, comparative cohorts should control for major confounders by reporting tumor complexity metrics (RENAL/PADUA), clamping strategy (global vs. selective/super-selective; arterial vs. segmental), and key baseline functional variables and should present renal outcomes using predefined, non-interchangeable timepoints (e.g., discharge, 1–3 months, and ≥6 months), ideally complemented by split renal function when available. Collectively, these minimum criteria would reduce heterogeneity, improve reproducibility, and allow more reliable synthesis across comparative cohorts and meta-analyses [8,11,12,56].

Third, progress in the fluorophore design and imaging depth is required. Current NIR-I imaging penetrates only a few millimeters, and at best up to about one centimeter, which is insufficient for deep or hidden lesions [70]. Emerging NIR-II and NIR-IIb imaging techniques offer enhanced microvascular visualization, structural and functional imaging at greater depth, and more favorable signal-to-noise characteristics and can support applications ranging from tumor delineation to navigation and phototherapy. In preclinical models, some NIR-II agents provide sustained fluorescence signals for 24 to 36 h after injection, making them less time-dependent than conventional NIR-I ICG [71]. Preclinical studies have shown the feasibility of NIR-II fluorescence imaging for intraoperative guidance in renal cell carcinoma models, clearly distinguishing between tumor and normal renal tissue using agents like ASP5354, which allows the visualization of tumor margins during partial nephrectomy in animal models. Moreover, extensive reviews of NIR-II fluorescence imaging highlight its utility in surgical navigation for malignancies, such as kidney tumors, and clarify the benefits of NIR-II over NIR-I in improving tissue penetration and tumor recognition during oncologic surgeries [72].

Targeted fluorophores, including antibody- or ligand-based tracers and nanoparticle carriers, may further improve the detection of complex or deeply situated tumors and enable more precise nephron-sparing approaches. For example, carbonic-anhydrase-IX-targeted sulfonamide probes have shown selective binding and NIR visibility in hypoxic tumor xenografts, and various ICG-loaded lipid, polymer, or mesoporous silica nanoparticles are being explored as both imaging agents and drug carriers with organ-specific accumulation profiles [73].

Finally, future work should better define context-specific application in special populations. Pediatric patients, pregnant individuals, older adults, and those with significant hepatic dysfunction may require tailored dosing and timing protocols owing to altered pharmacokinetics or increased vulnerability, and they remain underrepresented in existing ICG partial nephrectomy series. Integrating patient-specific clinical pathways and explicitly evaluating safety and performance in these groups will be essential to ensure safe and equitable implementation.

Advances in protocol standardization, quantitative imaging, next-generation fluorophores, and patient-specific clinical pathways will be crucial for ICG-guided partial nephrectomy to optimize renal preservation and deliver durable functional and oncologic outcomes.

## 7. Conclusions

Indocyanine green (ICG) near-infrared fluorescence (NIRF) imaging is an adjunct in partial nephrectomy and it provides real-time imaging of renal perfusion and vascular anatomy and may have potential to support tumor–parenchyma contrast in selected cases.

Current evidence is strong regarding the technical feasibility of the procedure and the ability of NIRF-ICG to aid selective or super-selective clamping. It provides reliable real-time visualization on tissue perfusion, which can help in identifying the limit between ischemic and perfused areas during tumor excision.

Controversially, in the clinical setting, the results of NIRF-ICG remain inconsistent when evaluating functional and oncological results. Some meta-analyses and comparative studies report small short-term differences in WIT and eGFR at early postoperative timepoints (these differences often do not persist during follow-up), while others do not show any superiority at all; most studies report no consistent differences in operative time, blood loss, complications, length of stay or positive surgical margins.

The EMERALD randomized controlled trial demonstrated no significant long-term functional advantage of ICG-guided super-selective clamping relative to conventional methods.

ICG has no specificity for benign or malignant tumors, it cannot distinguish between a solid renal cell carcinoma or an oncocytoma. It reflects perfusion/vascular anatomy rather than histological characteristics.

Future research must focus on the standardization of NIRF-ICG which has been obstructed by methodological inconsistency, dosage variability, timing administration and different imaging platforms. Complex renal masses or deeply endophytic tumors remain difficult to be evaluated with NIR-I fluorescence due to its penetration limit (2–5 mm).

For the standardization of NIRF-ICG technique, future research should concentrate on optimizing perioperative protocols, objectives, and quantifiable fluorescence measurements. The development of NIR-II imaging and other targeted tracers may help in overcoming current limits in depth and specificity.

## Data Availability

No new data were created or analyzed in this study.

## References

[1] Bex A., Ghanem Y.A., Albiges L., Bonn S., Campi R., Capitanio U., Dabestani S., Hora M., Klatte T., Kuusk T. (2025). European Association of Urology Guidelines on Renal Cell Carcinoma: The 2025 Update. Eur. Urol..

[2] Marconi L., Kuusk T., Hora M., Klatte T., Dabestani S., Capitanio U., Abu-Ghanem Y., Campi R., Fernández-Pello S., Albiges L. (2025). Hospital Volume as a Determinant of Outcomes After Partial Nephrectomy: A Systematic Review by the European Association of Urology Renal Cell Carcinoma Guidelines Panel. Eur. Urol. Oncol..

[3] McClintock T.R., Bjurlin M.A., Wysock J.S., Borofsky M.S., Marien T.P., Okoro C., Stifelman M.D. (2014). Can Selective Arterial Clamping with Fluorescence Imaging Preserve Kidney Function During Robotic Partial Nephrectomy?. Urology.

[4] Krane L.S., Manny T.B., Hemal A.K. (2012). Is Near Infrared Fluorescence Imaging Using Indocyanine Green Dye Useful in Robotic Partial Nephrectomy: A Prospective Comparative Study of 94 Patients. Urology.

[5] Dalli J., Jindal A., Gallagher G., Epperlein J.P., Hardy N.P., Malallah R., O’Donoghue K., Cantillon-Murphy P., Mac Aonghusa P.G., Cahill R.A. (2023). Evaluating clinical near-infrared surgical camera systems with a view to optimizing operator and computational signal analysis. J. Biomed. Opt..

[6] Zhou L., Zhou J., Shuai H., Xu Q., Tan Y., Luo J., Xu P., Duan X., Mao X., Wang S. (2024). Comparison of perioperative outcomes of selective arterial clipping guided by near-infrared fluorescence imaging using indocyanine green versus undergoing standard robotic-assisted partial nephrectomy: A systematic review and meta-analysis. Int. J. Surg..

[7] Giulioni C., Mulawkar P.M., Castellani D., De Stefano V., Nedbal C., Gadzhiev N., Pirola G.M., Law Y.X.T., Wroclawski M.L., Keat W.O.L. (2023). Near-Infrared Fluorescence Imaging with Indocyanine Green for Robot-Assisted Partial Nephrectomy: A Systematic Review and Meta-Analysis. Cancers.

[8] Gasperi A., De Mazza E., Prosperi M. (2016). Indocyanine green kinetics to assess liver function: Ready for a clinical dynamic assessment in major liver surgery?. World J. Hepatol..

[9] Li D., Smith B.D. (2021). Deuterated Indocyanine Green (ICG) with Extended Aqueous Storage Shelf-Life: Chemical and Clinical Implications. Chem.–A Eur. J..

[10] Lu C.-H., Hsiao J.-K. (2021). Indocyanine green: An old drug with novel applications. Tzu Chi Med. J..

[11] Wulf N.R., Schmitz J., Choi A., Kapusnik-Uner J. (2021). Iodine allergy: Common misperceptions. Am. J. Health-Syst. Pharm..

[12] Baig M. (2014). Shellfish allergy and relation to iodinated contrast media: United Kingdom survey. World J. Cardiol..

[13] Le-Nguyen A., O’Neill Trudeau M., Dodin P., Keezer M.R., Faure C., Piché N. (2021). The Use of Indocyanine Green Fluorescence Angiography in Pediatric Surgery: A Systematic Review and Narrative Analysis. Front. Pediatr..

[14] Chu W., Chennamsetty A., Toroussian R., Lau C. (2017). Anaphylactic Shock After Intravenous Administration of Indocyanine Green During Robotic Partial Nephrectomy. Urol. Case Rep..

[15] Papadia A., Luisa Gasparri M., DMueller M. (2017). Are allergic reactions to indocyanine green really that uncommon? A single institution experiences. Obstet. Gynecol. Rep..

[16] Yuan B., Chen N., Zhu Q. (2004). Emission and absorption properties of indocyanine green in Intralipid solution. J. Biomed. Opt..

[17] Vinegoni C., Botnaru I., Aikawa E., Calfon M.A., Iwamoto Y., Folco E.J., Ntziachristos V., Weissleder R., Libby P., Jaffer F.A. (2011). Indocyanine Green Enables Near-Infrared Fluorescence Imaging of Lipid-Rich, Inflamed Atherosclerotic Plaques. Sci. Transl. Med..

[18] Bae P.K., Jung J., Chung B.H. (2014). Highly enhanced optical properties of indocyanine green/perfluorocarbon nanoemulsions for efficient lymph node mapping using near-infrared and magnetic resonance imaging. Nano Converg..

[19] Chon B., Ghann W., Uddin J., Anvari B., Kundra V. (2023). Indocyanine Green (ICG) Fluorescence Is Dependent on Monomer with Planar and Twisted Structures and Inhibited by H-Aggregation. Int. J. Mol. Sci..

[20] Chen Q.-Y., Zhong Q., Liu Z.-Y., Li P., Lin G.-T., Zheng Q.-L., Wang J.-B., Lin J.-X., Lu J., Cao L.-L. (2023). Indocyanine green fluorescence imaging-guided versus conventional laparoscopic lymphadenectomy for gastric cancer: Long-term outcomes of a phase 3 randomised clinical trial. Nat. Commun..

[21] Fransvea P., Chiarello M.M., Fico V., Cariati M., Brisinda G. (2024). Indocyanine green: The guide to safer and more effective surgery. World J. Gastrointest. Surg..

[22] Wang R., Tang J., Chen Y., Fang Z., Shen J. (2021). The clinical value of indocyanine green fluorescence navigation system for laparoscopic partial nephrectomy in the case of complex renal clear cell carcinoma (R.E.N.A.L score ≥ 7). J. Cancer.

[23] Keller D.S., Ishizawa T., Cohen R., Chand M. (2017). Indocyanine green fluorescence imaging in colorectal surgery: Overview, applications, and future directions. Lancet Gastroenterol. Hepatol..

[24] Tobis S., Knopf J.K., Silvers C.R., Marshall J., Cardin A., Wood R.W., Reeder J.E., Erturk E., Madeb R., Yao J. (2012). Near Infrared Fluorescence Imaging After Intravenous Indocyanine Green: Initial Clinical Experience with Open Partial Nephrectomy for Renal Cortical Tumors. Urology.

[25] Diana P., Buffi N.M., Lughezzani G., Dell’Oglio P., Mazzone E., Porter J., Mottrie A. (2020). The Role of Intraoperative Indocyanine Green in Robot-assisted Partial Nephrectomy: Results from a Large, Multi-Institutional Series. Eur. Urol..

[26] Long J.-A., Fiard G., Giai J., Teyssier Y., Fontanell A., Overs C., Poncet D., Descotes J.-L., Rambeaud J.-J., Moreau-Gaudry A. (2022). Superselective Ischemia in Robotic Partial Nephrectomy Does Not Provide Better Long-Term Renal Function than Renal Artery Clamping in a Randomized Controlled Trial (EMERALD): Should We Take the Risk?. Eur. Urol. Focus..

[27] Pruimboom T., van Kuijk S.M.J., Qiu S.S., van den Bos J., Wieringa F.P., van der Hulst R.R.W.J., Schols R.M. (2020). Optimizing Indocyanine Green Fluorescence Angiography in Reconstructive Flap Surgery: A Systematic Review and Ex Vivo Experiments. Surg. Innov..

[28] Alius C., Gradinaru E.-S., Nica A.E. (2017). Indocyanine Green Fluorescence in Surgical Practice–A Narrative Review of Clinical Applications. Rom. J. Med. Pract..

[29] Papayan G., Akopov A. (2018). Potential of indocyanine green near-infrared fluorescence imaging in experimental and clinical practice. Photodiagnosis Photodyn. Ther..

[30] Fransvea P., Miccini M., Rondelli F., Brisinda G., Costa A., Garbarino G.M., Costa G. (2024). A Green Lantern for the Surgeon: A Review on the Use of Indocyanine Green (ICG) in Minimally Invasive Surgery. J. Clin. Med..

[31] Liu Z., Zeng T., Dang H., He H., Li J., Yan L., Yin D. (2025). Precision Visualization of Surgical Margins and Depth Imaging Performance in Orthotopic Hepatocellular Carcinoma Using Galactose-Targeted NIR-II Fluorescence Nanoprobe. Anal. Chem..

[32] Li Z., Li Z., Ramos A., Boudreaux J.P., Thiagarajan R., Mattison Y.B., Dunham M.E., McWhorter A.J., Chen Q., Zhang J. (2021). Detection of pancreatic cancer by indocyanine green-assisted fluorescence imaging in the first and second near-infrared windows. Cancer Commun..

[33] Cao C., Deng S., Wang B., Shi X., Ge L., Qiu M., Zhang F., Lu M., Ma L., Chi C. (2021). Intraoperative near-infrared II window fluorescence imaging-assisted nephron-sparing surgery for complete resection of cystic renal masses. Clin. Transl. Med..

[34] Dalli J., Gallagher G., Jindal A., Epperlein J.P., Hardy N.P., Malallah R., O’Donoghue K., Cantillon-Murphy P., Mac Aonghusa P.G., Cahill R.A., Elson D.S., Gioux S., Pogue B.W. (2022). Objective interrogation of signal presentation from surgical near-infrared fluorescence systems for user computerised interpretation. Clinical Biophotonics.

[35] Stolk D., Bloemen P., van den Elzen R.M., de Bruin M., Driessen C. (2025). A Potential Pitfall in the Interpretation of Microscope-Integrated Fluorescence Angiography: The Center–Periphery Effect. Med. Sci..

[36] Yoon K.-C., Kim K.G., Lee S.H. (2021). Design of a Surgical Pen-Type Probe for Real-Time Indocyanine Green Fluorescence Emission Diagnosis. J. Med. Devices.

[37] Chiti L.E., Park B., d’Orchymont F., Holland J.P., Nolff M.C. (2023). Impact of Surgical Lights on the Performance of Fluorescence-Guided Surgery Systems: A Pilot Study. Animals.

[38] Meltzer J.S. (2019). Renal Physiology. Pharmacology and Physiology for Anesthesia.

[39] Fasanella D. (2024). Intraoperative imaging techniques for robotic-assisted partial nephrectomy: Where do we stand?. Mini-Invasive Surg..

[40] Katsimperis S., Tzelves L., Bellos T., Manolitsis I., Mourmouris P., Kostakopoulos N., Pyrgidis N., Somani B., Papatsoris A., Skolarikos A. (2024). The use of indocyanine green in partial nephrectomy: A systematic review. Central Eur. J. Urol..

[41] Basatac C., Akpinar H. (2020). ‘Trifecta’ outcomes of robot-assisted partial nephrectomy: Results of the ‘low volume’ surgeon. Int. Braz. J. Urol..

[42] Bai N., Qi M., Shan D., Liu S., Na T., Chen L. (2022). Trifecta achievement in patients undergoing partial nephrectomy: A systematic review and meta-analysis of predictive factors. Int. Braz. J. Urol..

[43] Mjaess G., Bernhard J.-C., Khene Z.-E., Doumerc N., Vaessen C., Henon F., Bruyere F., Brenier M., Parier B., Albisinni S. (2023). Retroperitoneal vs. transperitoneal robotic partial nephrectomy: A multicenter propensity-score matching analysis (PADORA Study-UroCCR n° 68). Minerva Urol. Nephrol..

[44] Licari L.C., Bologna E., Proietti F., Flammia R.S., Bove A.M., D’annunzio S., Tuderti G., Leonardo C. (2023). Exploring the Applications of Indocyanine Green in Robot-Assisted Urological Surgery: A Comprehensive Review of Fluorescence-Guided Techniques. Sensors.

[45] Deka H., Mallikarjunarao Medam N., Kumar P.G.P.V., Gideon M., Ajith Kumar A., Prashanth Reddy Y., Barath Kumar S. (2024). Comparison of Trifecta and Pentafecta Outcomes across 3 Surgical Modalities of Partial Nephrectomy (PN)–Open, Lap, and Robotic. J. Kidney Cancer VHL.

[46] Manny T.B., Krane L.S., Hemal A.K. (2013). Indocyanine Green Cannot Predict Malignancy in Partial Nephrectomy: Histopathologic Correlation with Fluorescence Pattern in 100 Patients. J. Endourol..

[47] Warren H., Fanshawe J.B., Mok V., Iyer P., Chan V.W., Hesketh R., Zimmermann E., Kasivisvanathan V., Emberton M., Tran M.G.B. (2024). Imaging modalities for characterising T1 renal tumours: A systematic review and meta-analysis of diagnostic accuracy. BJUI Compass.

[48] Patel H.D., Johnson M.H., Pierorazio P.M., Sozio S.M., Sharma R., Iyoha E., Bass E.B., Allaf M.E. (2016). Diagnostic Accuracy and Risks of Biopsy in the Diagnosis of a Renal Mass Suspicious for Localized Renal Cell Carcinoma: Systematic Review of the Literature. J. Urol..

[49] Veccia A., Antonelli A., Hampton L.J., Greco F., Perdonà S., Lima E., Hemal A.K., Derweesh I., Porpiglia F., Autorino R. (2020). Near-infrared Fluorescence Imaging with Indocyanine Green in Robot-Assisted Partial Nephrectomy: Pooled Analysis of Comparative Studies. Eur. Urol. Focus.

[50] Panunzio A., Orlando R., Greco F., Cerrato C., D’Elia S.D., Marinaci L., Manno F., Shakir A., Battaglia M., Baccaglini W. (2025). The Role of Near-Infrared Fluorescence with Indocyanine Green in Robot-Assisted Partial Nephrectomy: Results from an Updated Systematic Review and Meta-Analyses of Controlled Studies. Medicina.

[51] Angell J.E., Khemees T.A., Abaza R. (2013). Optimization of Near Infrared Fluorescence Tumor Localization During Robotic Partial Nephrectomy. J. Urol..

[52] Yang Y.-K., Hsieh M.-L., Chen S.-Y., Liu C.-Y., Lin P.-H., Kan H.-C., Pang S.-T., Yu K.-J. (2022). Clinical Benefits of Indocyanine Green Fluorescence in Robot-Assisted Partial Nephrectomy. Cancers.

[53] Joffe B.I., Li G., Gorroochurn P., DeCastro G.J., Lenis A.T., McKiernan J.M., Anderson C.B. (2025). The impact of indocyanine green on partial nephrectomy perioperative outcomes. J. Robot. Surg..

[54] Thompson R.H., Lane B.R., Lohse C.M., Leibovich B.C., Fergany A., Frank I., Gill I.S., Blute M.L., Campbell S.C. (2012). Renal Function After Partial Nephrectomy: Effect of Warm Ischemia Relative to Quantity and Quality of Preserved Kidney. Urology.

[55] Ficarra V., Crestani A., Inferrera A., Novara G., Rossanese M., Subba E., Giannarini G. (2018). Positive Surgical Margins After Partial Nephrectomy: A Systematic Review and Meta-Analysis of Comparative Studies. Kidney Cancer.

[56] Dahlkamp L., Haeuser L., Winnekendonk G., von Bodman C., Frey U.H., Epplen R., Palisaar R.-J., Bach P., Noldus J., Brock M. (2019). Interdisciplinary Comparison of PADUA and R.E.N.A.L. Scoring Systems for Prediction of Conversion to Nephrectomy in Patients with Renal Mass Scheduled for Nephron Sparing Surgery. J. Urol..

[57] Buffi N.M., Saita A., Lughezzani G., Porter J., Dell’Oglio P., Amparore D., Fiori C., Denaeyer G., Porpiglia F., Mottrie A. (2020). Robot-Assisted Partial Nephrectomy for Complex (PADUA Score ≥ 10) Tumors: Techniques and Results from a Multicenter Experience at Four High-Volume Centers. Eur. Urol..

[58] Takahara K., Kusaka M., Nukaya T., Takenaka M., Zennami K., Ichino M., Sasaki H., Sumitomo M., Shiroki R. (2022). Functional Outcomes After Selective Clamping in Robot-Assisted Partial Nephrectomy. J. Clin. Med..

[59] Bove P., Bertolo R., Sandri M., Cipriani C., Leonardo C., Parma P., Falsaperla M., Veneziano D., Celia A., Mari A. (2021). Deviation from the Protocol of a Randomized Clinical Trial Comparing On-Clamp versus Off-Clamp Laparoscopic Partial Nephrectomy (CLOCK II Laparoscopic Study): A Real-Life Analysis. J. Urol..

[60] van Manen L., Handgraaf H.J.M., Diana M., Dijkstra J., Ishizawa T., Vahrmeijer A.L., Mieog J.S.D. (2018). A practical guide for the use of indocyanine green and methylene blue in fluorescence-guided abdominal surgery. J. Surg. Oncol..

[61] Paraboschi I., Farneti F., Jannello L., Manzoni G., Berrettini A., Mantica G. (2022). Narrative review on applications of fluorescence-guided surgery in adult and paediatric urology. AME Med. J..

[62] Son G.M., Ahn H., Lee I.Y., Ha G.W. (2021). Multifunctional Indocyanine Green Applications for Fluorescence-Guided Laparoscopic Colorectal Surgery. Ann. Coloproctol..

[63] Bishara A., Meir M., Portnoy E., Shmuel M., Eyal S. (2015). Near Infrared Imaging of Indocyanine Green Distribution in Pregnant Mice and Effects of Concomitant Medications. Mol. Pharm..

[64] Fineman M.S. (2001). Safety of Indocyanine Green Angiography During Pregnancy. Arch. Ophthalmol..

[65] Esposito C., Settimi A., Del Conte F., Cerulo M., Coppola V., Farina A., Crocetto F., Ricciardi E., Esposito G., Escolino M. (2020). Image-Guided Pediatric Surgery Using Indocyanine Green (ICG) Fluorescence in Laparoscopic and Robotic Surgery. Front. Pediatr..

[66] Esposito C., Lepore B., Cerulo M., Del Conte F., Coppola V., Esposito G., Carulli R., Carraturo F., Escolino M. (2023). Applications of indocyanine green (ICG) fluorescence technology in open surgery: Preliminary experience in pediatric surgery. Front. Surg..

[67] Jiao Y., Liu Y., Jin M. (2024). Exploring the dark side of diagnostic dyes with a focus on Indocyanine green’s adverse reactions. Sci. Rep..

[68] Pollmann L., Juratli M., Roushansarai N., Pascher A., Hölzen J.P. (2023). Quantification of Indocyanine Green Fluorescence Imaging in General, Visceral and Transplant Surgery. J. Clin. Med..

[69] Van Den Hoven P., Osterkamp J., Nerup N., Svendsen M.B.S., Vahrmeijer A., Van Der Vorst J.R., Achiam M.P. (2023). Quantitative perfusion assessment using indocyanine green during surgery—current applications and recommendations for future use. Langenbecks Arch. Surg..

[70] van der Vorst J.R., Schaafsma B.E., Verbeek F.P.R., Hutteman M., Mieog J.S.D., Lowik C.W.G.M., Liefers G.-J., Frangioni J.V., van de Velde C.J.H., Vahrmeijer A.L. (2012). Randomized Comparison of Near-infrared Fluorescence Imaging Using Indocyanine Green and 99m Technetium With or Without Patent Blue for the Sentinel Lymph Node Procedure in Breast Cancer Patients. Ann. Surg. Oncol..

[71] Luo Q., Tong Y., Li X., Zhang Z., Qin Y., Wang D., Tang B.Z. (2026). Covalent organic framework with second near-infrared aggregation-induced emission for boosted tumor multimodal phototheranostics. Biomaterials.

[72] Teranishi K. (2022). Near-Infrared Fluorescence Imaging of Renal Cell Carcinoma with ASP5354 in a Mouse Model for Intraoperative Guidance. Int. J. Mol. Sci..

[73] Janoniene A., Petrikaite V. (2020). In Search of Advanced Tumor Diagnostics and Treatment: Achievements and Perspectives of Carbonic Anhydrase IX Targeted Delivery. Mol. Pharm..

